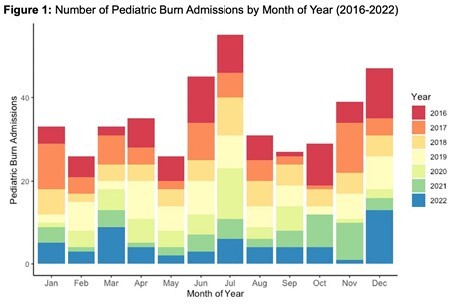# 47 Pediatric Burn Admissions: Where and When Matters

**DOI:** 10.1093/jbcr/irae036.071

**Published:** 2024-04-17

**Authors:** Erin E Ross, Justin Gillenwater, Haig A Yenikomshian

**Affiliations:** Keck School of Medicine of USC, Los Angeles, CA; Keck Medicine of USC, Los Angeles, CA; University of Southern California, Los Angeles, CA; Keck School of Medicine of USC, Los Angeles, CA; Keck Medicine of USC, Los Angeles, CA; University of Southern California, Los Angeles, CA; Keck School of Medicine of USC, Los Angeles, CA; Keck Medicine of USC, Los Angeles, CA; University of Southern California, Los Angeles, CA

## Abstract

**Introduction:**

There have been variable trends reported in seasonality of pediatric burns. Furthermore, risk for burn injury and mortality after burn have been associated with poorer socioeconomic status. The Childhood Opportunity Index (COI), comprised of 29 parameters of childhood well-being across the domains of Education, Health & Environment, and Social & Economic, may be a more comprehensive metric by which to assess rates of pediatric burn admission. Here, we explored trends in pediatric burn admissions seasonally, geospatially, and by COI quintile.

**Methods:**

After IRB approval, home zip code and date of injury for all pediatric burn admissions from 2016-2022 at an urban regional burn center. Each zip code in the US is assigned to a COI quintile from Q1 (very low) to Q5 (very high) opportunity, with a roughly equal number of children living in each quintile. Population of each zip code was obtained from US Census data. We assess the number of pediatric burn admissions by month of year and zip code, and risk for pediatric burn admission for children living in very high versus very low opportunity COI quintiles.

**Results:**

Across 470 pediatric burn admissions, there was a clear temporal trend with greater admissions in June, July and December, when children are out of school (Figure 1). Admissions for burn in children were also clustered in urban areas with higher population density (Figure 1). For all three COI sub-domains, there was an approximately five-fold increase in risk for admission for a burn for children residing in very low opportunity areas compared to children in very high opportunity areas. (Education OR 5.08 95% CI 3.54-7.27, Health & Environment OR 4.91 95% CI 3.34-7.12, Social & Economic OR 4.84 95% CI 3.41-6.86).

**Conclusions:**

Severe pediatric burns are clustered in areas with low educational, environmental, and socioeconomic opportunity. Given the temporal association burns with the school year and strong relationship of pediatric burns with educational opportunity, child supervision is a potential target for further study.

**Applicability of Research to Practice:**

Identifying community-level risk factors and region-specific temporal trends in pediatric burn injury can help identify highest yield areas for education and injury prevention efforts.